# To Eat Well or to Live Ill

**Published:** 1921-03-26

**Authors:** 


**THE PUBLIC WELL-BEING**—(*continued*).

### To Eat Well or to Live Ill.

In the memorandum which the Ministry of Health is being urged to distribute to the public on the subject of healthy diet the official compiler could not do better than take as his basis the pamphlet recently prepared by Dr. Eric Pritchard and issued by the People's League of Health, whose energetic and judicious propaganda in numerous directions of health reform cannot be too highly praised. In many respects Dr. Pritchard's observations cross the t's and dot the i's of our own comments on this subject in a recent issue. He points out that mistakes in diet are responsible for an immense amount of sickness and disease, and that they occur as the result of ignorance more than as the result of economic or social conditions. It is too commonly believed that correct dietaries can be arranged by a simple compliance with taste and customs. Many people appear to believe that scientific adjustments, based on definitely ascertained principles, are useless and unnecessary. Until, however, the scientific study of dietaries and dietetics is more seriously taken up by the medical and nursing professions the populace, as a whole,

**THE PUBLIC WELL-BEING**—(*continued*).

will continue to suffer from many unnecessary forms of malnutrition. We are glad to see that in this lucid and convincing little pamphlet stress is laid on the importance of the psychological factor in diet—a point which is generally overlooked. It is in one-sided and monotonous dietaries that the consumer is apt to suffer very seriously and to develop some deficiency disease or symptom.

There can be little doubt, as Dr. Pritchard asserts, that a large section of our population, particularly the urban part, receives great damage from an inadequate supply of fresh food, especially of green vegetables, raw salads, and fruit, and that they exist too exclusively on preserved foods, meat, and highly refined flour. Moreover, " tastes for rich and unwholesome foods, as well as antipathies for wholesome articles of diet, are very easily acquired by children through indiscreet remarks and actions on the part of parents, nurses, and others who manage or associate with them. Likes and dislikes of food are practically always acquired and perpetrated by ' suggestion '; they are not inherited. It is true that there are idiosyncrasies of a definitely physical kind which certain individuals possess in respect of particular kinds of food; for instance, some people cannot eat shellfish, almonds, or strawberries without suffering from severe symptoms of a constitutional kind or developing nettle rash or other skin troubles." But these are rare exceptions, which do not affect the main argument. If the suggested Government memorandum is to serve a really useful service it must show in the simplest and clearest, and therefore most convincing, form that for a diet to be physiological it must fulfil definite requirements in connection with " caloric value, balance, digestibility, times of consumption, and certain accessory and psychological factors. The whole subject assumes real and vital importance, affecting the happiness of every family in the kingdom, the richest as well as the poorest, when it is realised that the whole of life, a man's policy in public and private life, " can be entirely altered by dyspeptic conditions due to dietetic indiscretions."

